# SE-enhanced 1-D CNN with full-band hyperspectral imaging for rapid and accurate maize seed variety classification

**DOI:** 10.3389/fpls.2025.1587845

**Published:** 2025-10-08

**Authors:** Linzhe Zhang, Chengzhong Liu, Junying Han, Kai Sun, Yongqiang Feng

**Affiliations:** College of Information Science and Technology, Gansu Agricultural University, Lanzhou, China

**Keywords:** hyperspectral imaging technology, maize seeds, classification, convolutional neural network, full band

## Abstract

**Introduction:**

Accurate identification of maize seed varieties is essential for enhancing crop yield and ensuring genetic purity in breeding programs.

**Methods:**

This study establishes a non-destructive classification approach based on hyperspectral imaging for discriminating 30 widely cultivated maize varieties from Northwest China. Hyperspectral images were acquired within the 380–1018 nm range, and the embryo region of each seed was selected as the region of interest for spectral extraction. The collected spectra were preprocessed using Savitzky–Golay (SG) smoothing. Several machine learning models—KNN, ELM, and a two-layer convolutional neural network integrated with squeeze-and-excitation (SE) attention modules (CNN2c-SE)—were constructed and compared.

**Results:**

Results demonstrated that the CNN2c-SE model utilizing full-spectrum data achieved a superior classification accuracy of 93.89%, significantly outperforming both conventional machine learning models and feature-waveband-based approaches.

**Discussion:**

The proposed method offers an effective and efficient tool for high-throughput, non-destructive maize seed variety identification, with promising applications in seed quality control and precision breeding.

## 1 Introduction

Corn is one of the world’s three major food crops and is extensively cultivated in Northwest China. It serves as a crucial food source and economic asset for the local population. Scientific cultivation practices aimed at increasing maize yield can provide a stable income for local communities and ensure a reliable and secure food supply (Wang et al., 2021). The scientific cultivation of corn can bring stable income to local residents and ensure a safe and stable supply of food. At present, there are many varieties of maize planted in Northwest China, the accurate identification of maize varieties is the basis for cultivating suitable maize varieties, which is also the key to realize high yield of maize, and at the same time, it can facilitate the cultivation and management of maize for the farmers and facilitate the improvement of excellent varieties. Therefore, it is of great significance to quickly and accurately categorize different varieties of maize.

Traditional methods of maize variety classification mainly include manual and biological detection methods, such as DNA sequencing, SNP analysis, etc (Zhou et al., 2022). There are difficulties in accurately identifying and subjectivity in manual testing, while biological testing methods require professional knowledge, high costs, and are prone to damage to seeds. Hyperspectral technology can identify seeds quickly and non-destructively to meet the production needs of modern agriculture. Due to the varying external structures and internal substances within seeds, light undergoes both penetration and interaction. Consequently, spectral reflectance curves represent not merely surface properties but composite signals carrying the molecular composition fingerprint of the seed’s interior. Differences in the concentration and composition of internal biochemical components—such as starch, protein, lipids, and moisture content—between seed varieties directly alter their unique absorption patterns, thereby modifying the reflected signals we measure. Hyperspectral imaging technology reflects the internal characteristics of seeds in the spectral curves, which can be used for rapid and non-destructive classification and identification, and is widely used in the detection of varieties (Sun et al., 2025), quality and vigor of various types of seeds (Zhang et al., 2022). Yang et al. used hyperspectral imaging to collect germ-side and endosperm-side images of four types of waxy maize, and used SG smoothing and continuous projection algorithms for preprocessing and feature wavelength selection, respectively, and used SVM and PLS-DA as the classification models, and the classification accuracies of SVM on the germ-side and the endosperm-side were 98.2% and 96.3%, which were higher than that of the PLS-DA model (Yang et al., 2015). Zhang et al. used hyperspectral imaging technology to collect images of three kinds of maize seeds with different degrees of frost damage, and used four kinds of processing methods, four kinds of feature wavelength selection methods and three different classification methods to build the model, and found that SG smoothing preprocessing, full-waveband PLS-DA had the highest accuracy and the best effect of the classification model (Zhang et al., 2019). Collins et al. used hyperspectral technology to collect images of viable and non-viable two maize seeds in the range of 1000–2500 nm, and used three models, LDA, PLS-DA, and SVM, as classification models, with the SVM model having the highest classification accuracy (Wakholi et al., 2018). Ji et al. proposed a combination of hyperspectral imaging technology and multi-classification support vector machine to classify six different potatoes, which was modeled by LDA dimensionality reduction and SVM, and the potatoes not involved in the modeling were segmented using K-means clustering, and after segmentation, the spectra of the regions were extracted and input into the model for recognition, with an accuracy of up to 90% (Ji et al., 2019). Therefore, the use of hyperspectral imaging technology to collect images, extract the feature wavelength using traditional machine learning modeling methods can be effective in the classification of various seeds in agricultural production.

In recent years, deep learning methods have been widely used in computer vision, natural language processing, smart agriculture, etc (Yan et al., 2021). Zhang et al. used hyperspectral imaging and DCNN model to classify four different corn varieties, and the accuracy of both test and validation sets was higher than that of KNN and SVM classification models, reaching 94.4% and 93.3%, respectively (Zhang et al., 2021). Xu et al. used hyperspectral imaging to build a CNN-FES model for feature wavelength selection, and a CNN-ATM model to classify healthy maize seeds and maize seeds suffering from worms, and the accuracy, sensitivity and specificity could reach 97.50%, 98.28% and 96.77%, respectively (Xu et al., 2023). Zhang et al. used hyperspectral imaging to acquire 735 corn seed images of seven semi-hybrid corn varieties in the range of 900–1700 nm, and established a (SG+MN)-(CARS+SPA)-CNN classification model with an accuracy of 96.65% by using different preprocessing methods and feature selection methods (Zhang et al., 2023). Wang et al. used combined hyperspectral imaging to obtain images of wheat flour with high content of deoxynivalenol and normal wheat flour, and combined the preprocessing methods and machine learning classification models to obtain the SG-CARS-RF model, which had the highest recall of 98.95%, and the accuracy of classification using the CNN model was higher at 97.78% (Wang et al., 2024). Seo et al. used distilled water to dilute potato juice and spinach juice to six different concentration levels and captured images through visible and near-infrared hyperspectral imaging, and used six classification models to classify them separately, and the CNN was the most effective, which was able to achieve 99% and 98%, respectively (Seo et al., 2021). Therefore, it is feasible to use deep learning models for classification (Zhao et al., 2026).

At present, there are a large number of domestic and international studies on the classification and identification of maize varieties, and most of the studies first carry out the selection of feature wavelengths, and then classification is performed using either traditional machine learning methods or deep learning methods as classification models (Cui et al., 2020). Traditional Machine Learning is usually based on statistical principles and has strong interpretability, such as KNN, ELM and other methods as classification models (Somvanshi et al., 2016). In contrast, deep learning models such as convolutional neural networks (CNNs) can automatically learn hierarchical features directly from raw or preprocessed data, integrating feature extraction and classification within a single learning framework, and perform well on complex tasks (LeCun et al., 2015). Most studies rely on traditional methods such as PLA and SPA for extracting feature wavelengths, which can result in the loss of detailed information and involve manual intervention. Additionally, many existing maize variety classification tasks use a limited number of varieties, making it challenging to achieve effective results in classifying a wide range of maize species. Given the extensive diversity of maize varieties, these limitations can impact the accuracy and reliability of classification outcomes (Kang et al., 2022). In this study, 30 maize varieties were selected and their full-band spectral information was used for the classification task, which ensured that the full-band information would not be lost, improved the utilization of features (Jia et al., 2023). This ensures that the whole operation is done by the machine, which reduces the impact caused by human factors and simplifies the operation to save time.Spectral preprocessing is a critical step in hyperspectral data analysis to enhance the useful chemical information and minimize the influence of various physical and instrumental artifacts. Raw spectral data often contain unwanted noise from the sensor electronics, light scattering effects due to sample morphology, and baseline shifts. Therefore, this study employs polynomial smoothing (SG), Gaussian filtering (GF), median filtering (MF), and moving average filtering (MA) as preprocessing methods to process the raw spectral data. In this study, CNN2c-SE, a CNN network model based on the attention mechanism, was established for classifying different maize varieties, and its classification accuracy was compared with that obtained from the machine learning models KNN and ELM, as well as different feature band selection methods.

## 2 Materials and methods

### 2.1 Experimental materials

**Figure 1:**
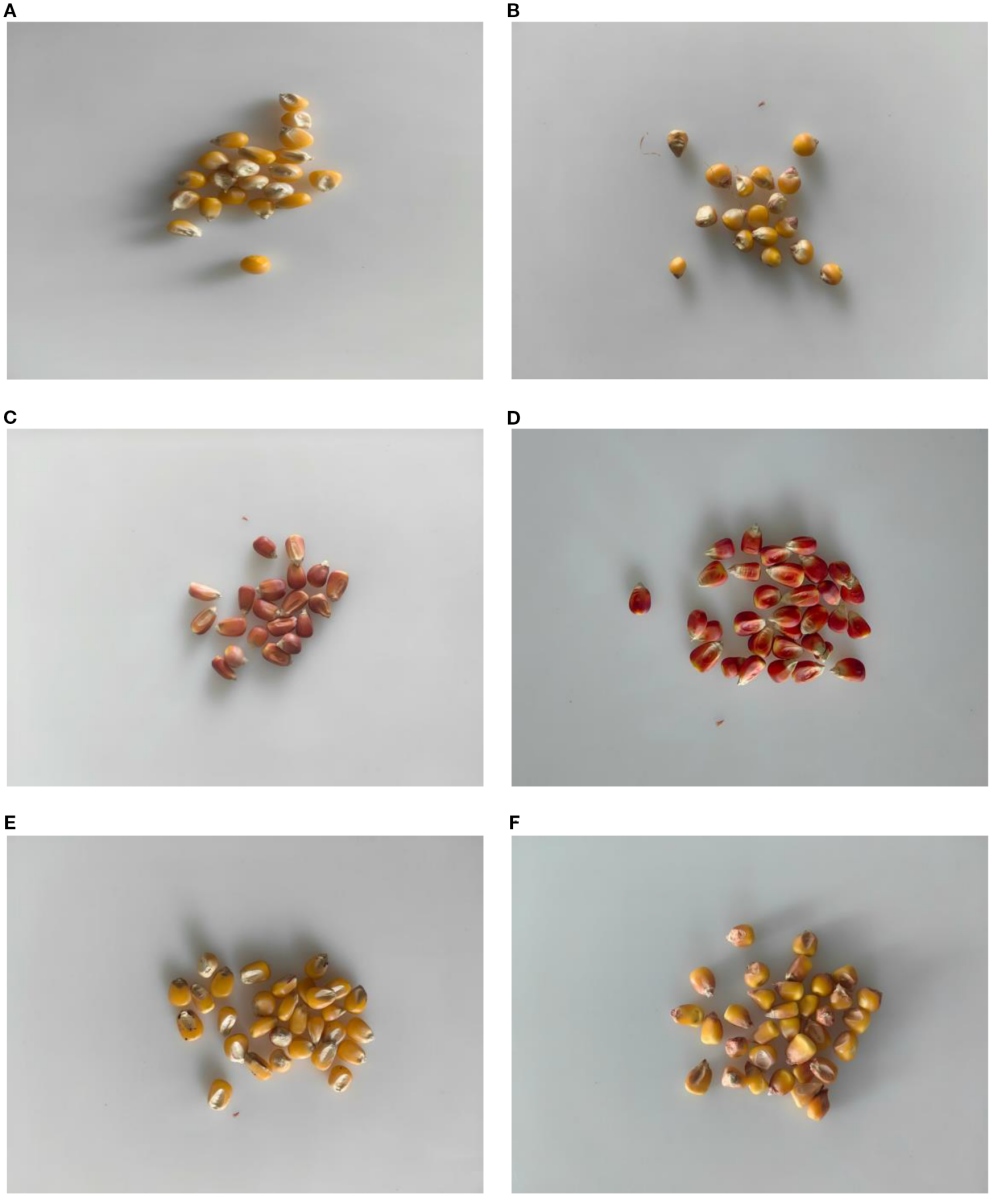
Representative Corn Seed Diagram. The **(A)** is JAM1910, No. 15. The **(B)** is QBFY166, No. 27. The **(C)** is QAGNY599, No. 13. The **(D)** is GMBSB1259, No. 11. The **(E)** is ZHAXY99, No. 21. The **(F)** is ZBQF218, No. 12.

The maize seeds used in this experiment were all provided by the Gansu Provincial Academy of Agricultural Sciences (GSAAS), and 30 varieties of maize seeds widely planted in Northwest China were selected, such as Early A Shengyuan 688 (ZASY688), Zhongshui A Shijiang (ZSASQ66), Zhongshui A Longxin District 518 (ZSALXQ518), and Early B Songyu 438 (ZQBSY438), etc, which were labeled from 1 to 30, respectively. In order to avoid the effect of moisture in the air, the corn seeds were stored in sealed paper bags, and 30 intact and unbruised corn seeds were selected for hyperspectral image collection at each sampling. The maize varieties are shown in **Table 1**. Representative seed images are shown in **Figure 1**.

**Table 1:** Corn varieties.

Name	No.	Number	Name	No.	Number	Name	No.	Number
ZASY688	1	30	GMBSB1259	11	30	ZHAXY99	21	30
ZSASQ66	2	30	ZBQF218	12	30	ZHBP220	22	30
ZSALXQ518	3	30	QAGNY599	13	30	ZSBJYY1207	23	30
ZQBSY438	4	30	JBL3712	14	30	ZSAZT2208	24	30
ZSPY128	5	30	JAM1910	15	30	QBFLQC2	25	30
ZAQF216	6	30	ZSDTY71110	16	30	JAC1219	26	30
ZBHT526	7	30	ZAQKY918	17	30	QBFY166	27	30
ZBJL6	8	30	GADF1908	18	30	ZAZD1568	28	30
GBJH25	9	30	JAGNY168	19	30	ZSBZT2209	29	30
ZJNY516	10	30	ZBNF7019	20	30	QBFLQC5	30	30

### 2.2 Experimental equipment

**Figure 2:**
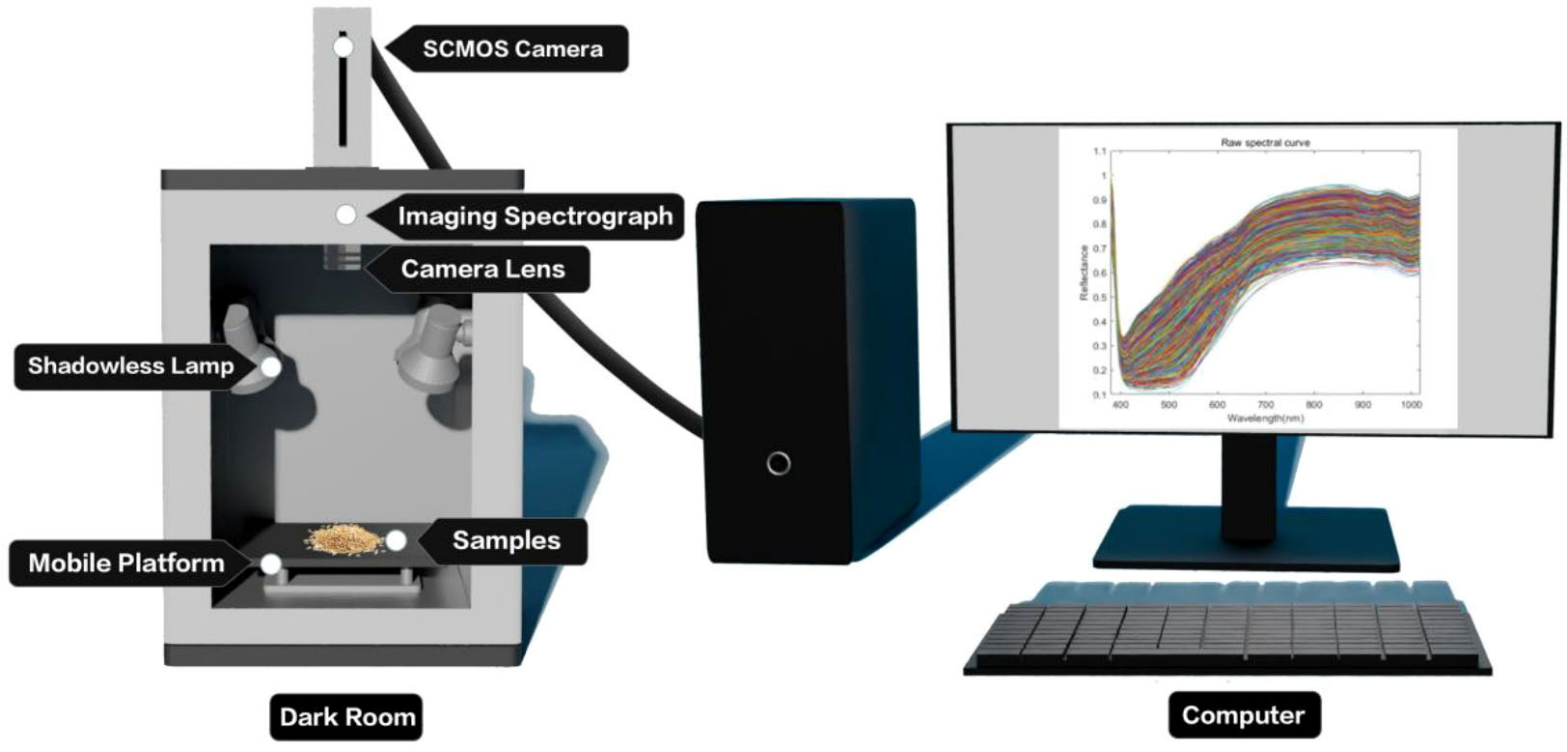
Hyperspectral imaging system.

The Gaia Field portable hyperspectral system (Sichuan Dualix Spectral Imaging Technology Co., Ltd.) is shown in **Figure 2**, which includes a GaiaField-V10E hyperspectral camera, a 2048×2048 pixel imaging lens, a HSI-CT-150×150 standard whiteboard (PTFE), a HSIA-DB indoor imaging dark box, four sets of shadowless lamp light sources, a HSIA- TP-L-A tripod rocker set, and hyperspectral data acquisition software Spec View. The spectral range is 380–1018 nm, the spectral band is 320, the spectral resolution is 2.8 nm, the numerical aperture is F/2.4, the slit size is 30 mm × 14.2 mm, the detector is SCMOS, the imaging modes are built-in push-scan, autofocus, and the dynamic range is 14 bits. The core components of the hyperspectral equipment include a standardized light source, a spectral camera, an electronically controlled mobile platform, a computer and a control software. The system works by using a push-scan imaging mode that combines a surface array detector with an imaging spectrometer. Driven by the scanning-controlled motorized platform, the slit of the imaging spectrometer and the focal plane of the imaging lens move relative to each other, and the detector collects real-time information relative to the line target, which is finally stitched together into a complete data cube.

### 2.3 Acquisition and correction of hyperspectral images.

**Figure 3:**
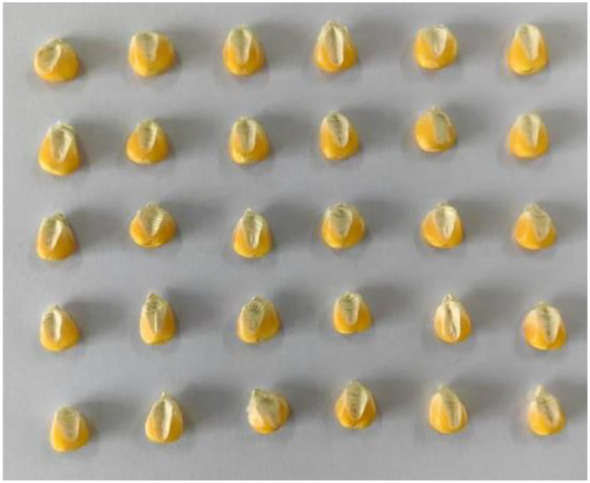
Corn seed placement chart.


Before image acquisition, the hyperspectral instrument switch and dark box light source were set. A warm-up time of 30 min was allowed, and then the instrument parameters were configured to set the camera exposure time to 49 ms, the gain to 2, the frame rate to 18.0018 Hz, and the forward speed to 0.00643 cm/s. Corn seed embryos are rich in nutrients, such as starch and proteins, and therefore, in this experiment, we collected image information of the embryonic surfaces of the samples. We chose a total of 30 maize seed varieties; for each hyperspectral image was collected a total of three times, each time 30 seeds were randomly selected from the corresponding varieties and embryo-surface-facing were placed in a dark box on the mobile platform for collection. After each variety was collected once, the sample under test was re-poured into the sample bag and manually shaken evenly. Then, 30 seeds were randomly selected and arranged in a 5×6 grid, as shown in **Figure 3**, for subsequent image acquisition of this variety. Each variety was repeated three times, resulting in a total of 90 seeds scanned. A total of 2,700 seeds were scanned, yielding 2,700 spectral curves. In order to improve the stability and reliability of the images, after the acquisition was completed, the original hyperspectral images were corrected in black and white to eliminate the effects caused by dark current noise (Polevoi et al., 2023). The formula for black and white correction is shown in Equation 1:

(1) $$I_{raw}=\frac{I_{raw}-I_{dark}}{I_{white}-I_{dark}}$$

Where I_raw_ is the original image, I_white_ is the white reference image, I_dark_ is the dark reference image and I_c_ is the calibration image.

To extract spectral information from the corrected hyperspectral images, spectral features were extracted using ENVI5.3 software, by intercepting the germ portion of each corn seed in a single image with a rectangle of the same size as the region of interest. Establish the obtained spectral curve as a Spectral Library, extracting the corresponding ASCII values for each wavelength band as experimental data.

### 2.4 Spectral pre-processing

In the process of image acquisition with hyperspectral instruments, it is inevitable to be interfered by instrumental noise, environmental noise, etc. In order to reduce the interference of irrelevant information, improve the quality of the obtained data, so as to ensure that the established model obtains more accurate prediction, it is necessary to carry out the preprocessing of the spectral data. In this study, polynomial smoothing (SG), Gaussian filter (GF), median filter (MF), and moving average filter (MA) are used as preprocessing methods to preprocess the original spectral data. SG smoothing is a commonly used data smoothing method, which is mainly used for smoothing the signal. The method achieves smoothing and preserves the features of the data by fitting polynomials to localized regions of the data points. The polynomial smoothing method (SmoothingSGolay) is characterized by its ability to smooth the trend of the data and remove the noise efficiently while keeping the overall shape and characteristics of the data intact (Hearst et al., 1998). Gaussian Filter Gaussian Filter Method (Gaussian Smoothing) is a commonly used smoothing method in image processing and signal processing. The method uses a Gaussian function as a weighting function, which is weighted and averaged over a localized area to achieve smoothing of a signal or image. The Gaussian filter method is effective in smoothing the signal while maintaining the image details and has good suppression of noise (Ala-Luhtala et al., 2014). Median Filtering. MedianFilter is a nonlinear smoothing technique mainly used for image denoising. Its basic principle is to set the grayscale value of each pixel point to the median of the grayscale values of all pixel points within a certain neighborhood window of that point. Specifically, median filtering is done by some sort of structured 2D sliding template, where the pixels within the template are sorted according to the magnitude of the pixel value, and then the median value is taken as the value of the current pixel point. This method helps to eliminate isolated noise points and bring the surrounding pixel values closer to the true values (Lee, 2019). MovingAverage filtering is commonly used in the processing of time series data or one-dimensional signals to eliminate short-term fluctuations and noise and to highlight long-term trends or cyclical changes. The basic idea is to take the average of the data points in a certain range before and after each data point in the time series data as the filtering result of that point (Dicle and Levendis, 2017).

### 2.5 Division of training and test sets

This study forms hyperspectral data cubes corresponding to the reflectance of each seed in each band, which serve as the final data input. In this experiment, 2700 samples were divided into training and test sets in the ratio of 4:1, and the training and test sets for each maize seed were 72 and 18, respectively.The training and test sets for the 30 species were 2160 and 540, respectively, in order to analyze and compute the discriminative accuracy of the training and test sets of the model. The experiment employed 10-fold cross-validation to reduce overfitting.

### 2.6 Experimental environment

**Figure 4:**
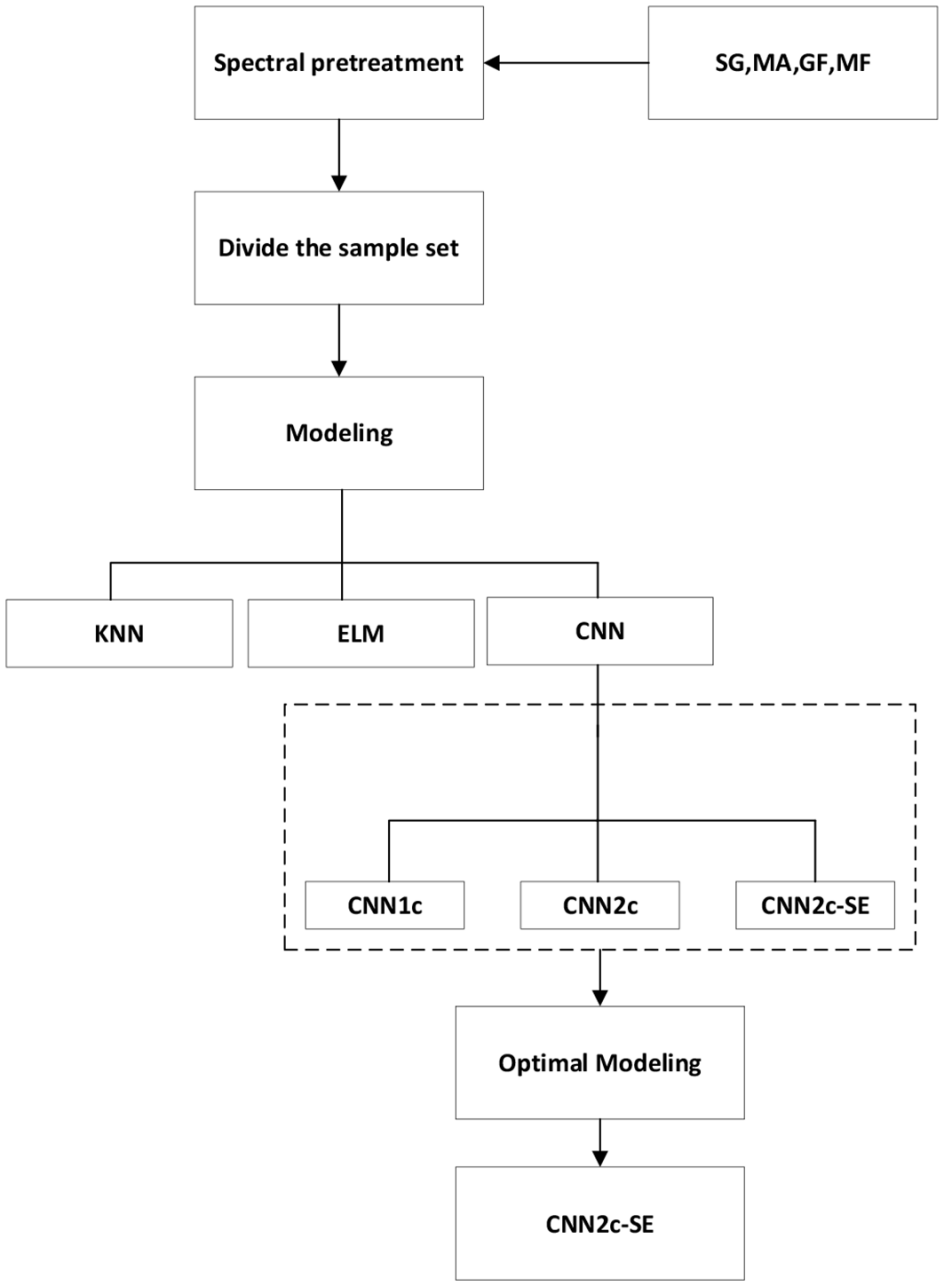
Flowchart of the experiment.

The machine learning model code was implemented using Matlab R2023a, and the deep learning model code was developed and researched using Python programming language, Pytorch environment. The running environment of the model is Windows 11 system, and the hardware information is as follows: CPU:13th Gen Intel(R) Core(TM) i7-13700 2.10 GHz; GPU: NVIDIA GeForce RTX 4090.The whole experimental process is shown in **Figure 4**.

## 3 Modeling

### 3.1 Machine learning models

K-Nearest Neighbors (KNN) is a commonly used classification algorithm that is based on the principle of proximity and makes classification or regression predictions by finding the K nearest training set samples to the sample to be classified (Yu et al., 2010). (1) The KNN algorithm uses feature vectors to represent the characteristics of each sample. These features can be any number and type of numerical, categorical, or textual data. (2) The similarity between the samples is measured by calculating the distance between them. (3) For a sample to be classified, the KNN algorithm calculates the distance between it and each sample in the training set and selects the K neighboring samples closest to it. Based on the categories of the K neighboring samples, the category of the sample to be classified is determined by majority voting. In this study, the data is first preprocessed using mean processing, maximum and minimum value processing and logarithmic processing. Next the standard Euclidean distance, correlation distance, Mahalanobis distance and other metrics were used and the value of K was set from 1 to 20, comparing the results for different values of k and determining K = 17.

Extreme Learning Machine (ELM) is a fast learning algorithm based on a single hidden layer feed forward neural network (Ding et al., 2014). Compared with traditional neural network algorithms, ELM randomly initializes the connection weights and biases between the input layer and the hidden layer without the iterative training process of back-propagation, the parameters of the hidden layer can be directly generated randomly, and only the weights of the output layer need to be trained. ELM can obtain the global optimal solution by performing the least-squares method on the randomly initialized hidden layer, avoiding the problem of going to the local optimal solution in traditional neural networks. ELM can use hidden layers with nonlinear activation functions to enhance the expressive power of the model and its training speed is fast enough to handle large-scale datasets. In this study, the Sigmoid function is used as the activation function, and the number of nodes in the hidden layer is set to 100, 150, and 200, respectively, and the number of nodes in the hidden layer is set to 150 after comparing the different results.

### 3.2 Deep learning models

**Figure 5:**
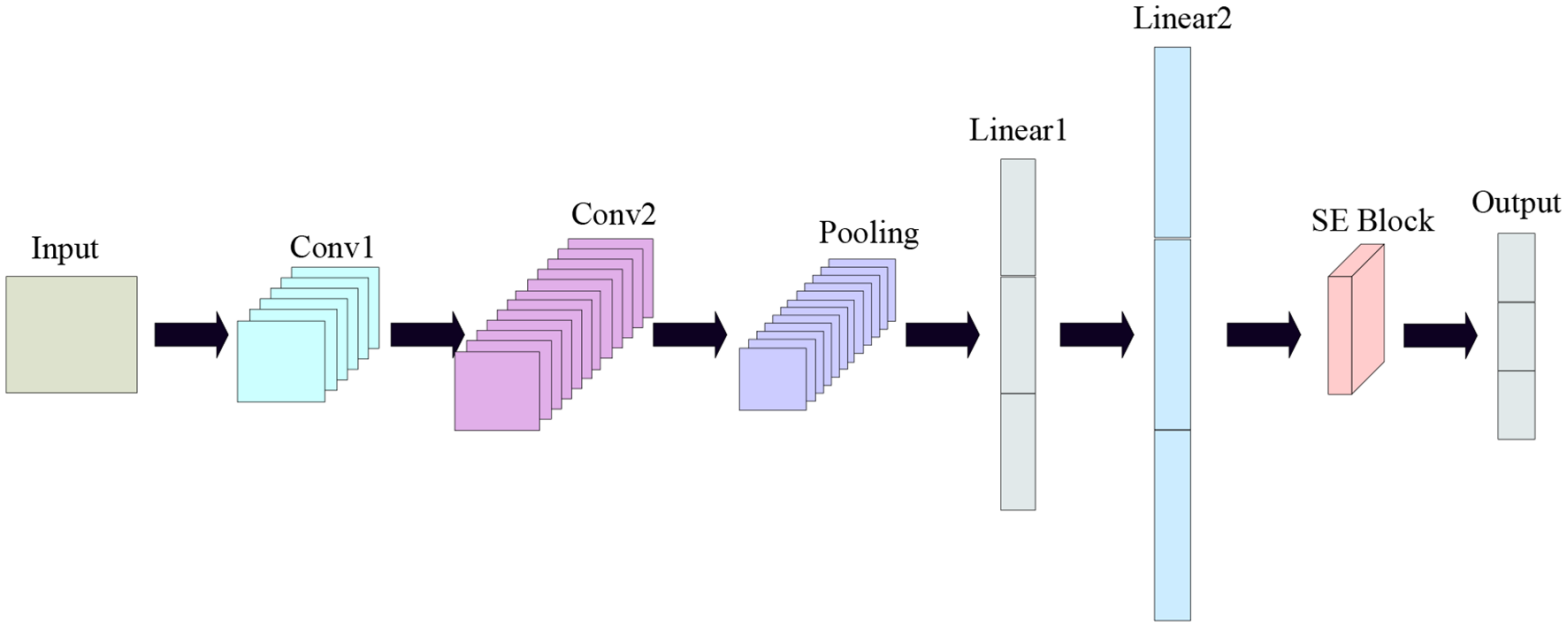
Convolutional neural network diagram.

Convolutional Neural Network (CNN) is a deep learning model that is mainly used to process data with a grid structure, such as images, text, video and sound (Ma et al., 2012). CNN had great success in computer vision tasks such as image classification, object detection, and image generation (Yeh et al., 2018). Its network structure is shown in **Figure 5**.In this experiment, one, two and three layers of convolutional and fully connected layers were experimented respectively, and it was found that two layers of convolutional and fully connected layers had the best classification effect, so the convolutional neural network in this experiment uses two layers of convolutional layers and two layers of fully connected layers.

**Figure 6:**
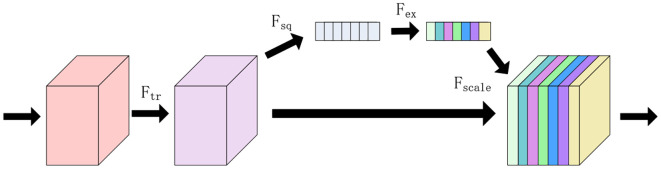
SE block structure.

In this study, a new one-dimensional CNN network model is proposed, which consists of one input layer, two convolutional layers, two fully-connected layers, one SEBLOCK module and one output layer. In this study, all 320 wavelength data are used as input data, and kernel_size, stride, and padding are set to 3,1,1, respectively, the initial learning rate is set to 0.01, and the batch_size is set to 64, and a total of 10,000 iterations are performed. the SE Block (SQUEEZE-AND- EXCITATION BLOCKS) module: the SE Block mainly contains two parts, Squeeze and Excitation (Mou et al., 2023). Its core idea is to learn the feature weights through the network according to the LOSS, which makes the Effective feature maps have bigger weights and the ineffective or less effective feature maps have smaller weights to train the model to achieve better results (Wang et al., 2022). The structure is shown in **Figure 6**.

Considering F_tr_ as a simple convolution operation, denoted by the equation V = [v_1_,v_2_,…,v_c_], where v_c_ denotes the cth convolution kernel, and the output is denoted by the equation U_c_ = [u_1_,u_2_,…,u_c_], related formulas is shown in Equation 2:


(2) $$u_{c}=v_{c}*X=\sum_{s=1}^{C^{'}}v_{c}^{s}*x^{s}$$

where * denotes convolution, v_c_ = $\left[\text{v}1_{\text{c}},{\text{v}2}_{\text{c}},\ldots ,{\text{vc"}}_{\text{c}}\right]$ , X=[x^1^,x^2^,…,x^c^].

Squeeze part: the Squeeze operation is to compress each feature map after obtaining multiple feature maps using a global average pooling operation, so that its C feature maps end up as a 1 × 1 × C array of real numbers (S. et al., 2022).

Excitation section: a simple gating mechanism with sigmoid activation is used to fully capture channel dependencies.

RELU function: this study uses the RELU function as the activation function, which is a nonlinear function that enhances the nonlinear relationship between the layers of the neural network compared to traditional neural network activation functions, such as the sigmoid function and the tanh function, which enhances the network’s ability to learn and express itself, and more efficiently Gradient descent and backpropagation, accelerate the training speed of neural network, can effectively solve the problem of gradient vanishing, will also make the output of neural network has sparsity, reduce overfitting phenomenon (He et al., 2022).

ADAM Optimizer: In this study, network optimization is performed using the ADAM optimizer, which is an adaptive optimization algorithm that adjusts the learning rate based on historical gradient information, and achieves faster convergence and better generalization ability by calculating a different adaptive learning rate for each parameter. It allows the neural network to use a larger learning rate in the early stage of training to converge quickly, and a smaller learning rate in the later stage of training to find the minimum of the loss function more accurately, avoiding overlearning the training data and thus reducing the risk of overfitting. Compared to other optimization algorithms, such as Adagrad and RMSProp, the Adam optimizer performs better when dealing with non-smooth objective functions (Zhang et al., 2023).

### 3.3 Model evaluation indicators

The classification of maize varieties in this study is a multi-category classification task with the same number of samples in each category, the accuracy rate is applicable to a dataset with a balanced distribution of categories, and is able to indicate the number of correctly classified samples as a proportion of the total number of samples. Therefore, this study employs Accuracy, Precision, Recall, and F1 score as model evaluation metrics (Zhao, 2012). Their calculation formulas are shown in the Equations 3–6). Where TP is the true example, FN is the false negative example, FP is the false positive example and TN is the true negative example.


(3) $$Accuracy=\frac{TP+TN}{TP+TN+FP+FN}$$

(4) $$\mathrm{Pr}ecision=\frac{TP}{TP+FP}$$

(5) Recall=TPTP+FN

(6) $$\text{F}1=2\times \frac{\text{Precision}\times \text{Recall}}{\text{Precision}+\text{Recall}}$$

In this study, cross entropy is used as the loss function of the model, which focuses on the degree of discrepancy between the predicted and true values of the model, and is fast to learn when the model is less effective, it is suitable for multiclassification models (Jamin and Humeau-Heurtier, 2020). The loss function formula is shown in Equation 7.


(7) $$Loss=-\frac{1}{N}\sum_{i}\sum_{c=1}^{M}y_{ic}\log(p_{ic})$$

## 4 Results and analysis

### 4.1 Spectral preprocessing results

**Figure 7:**
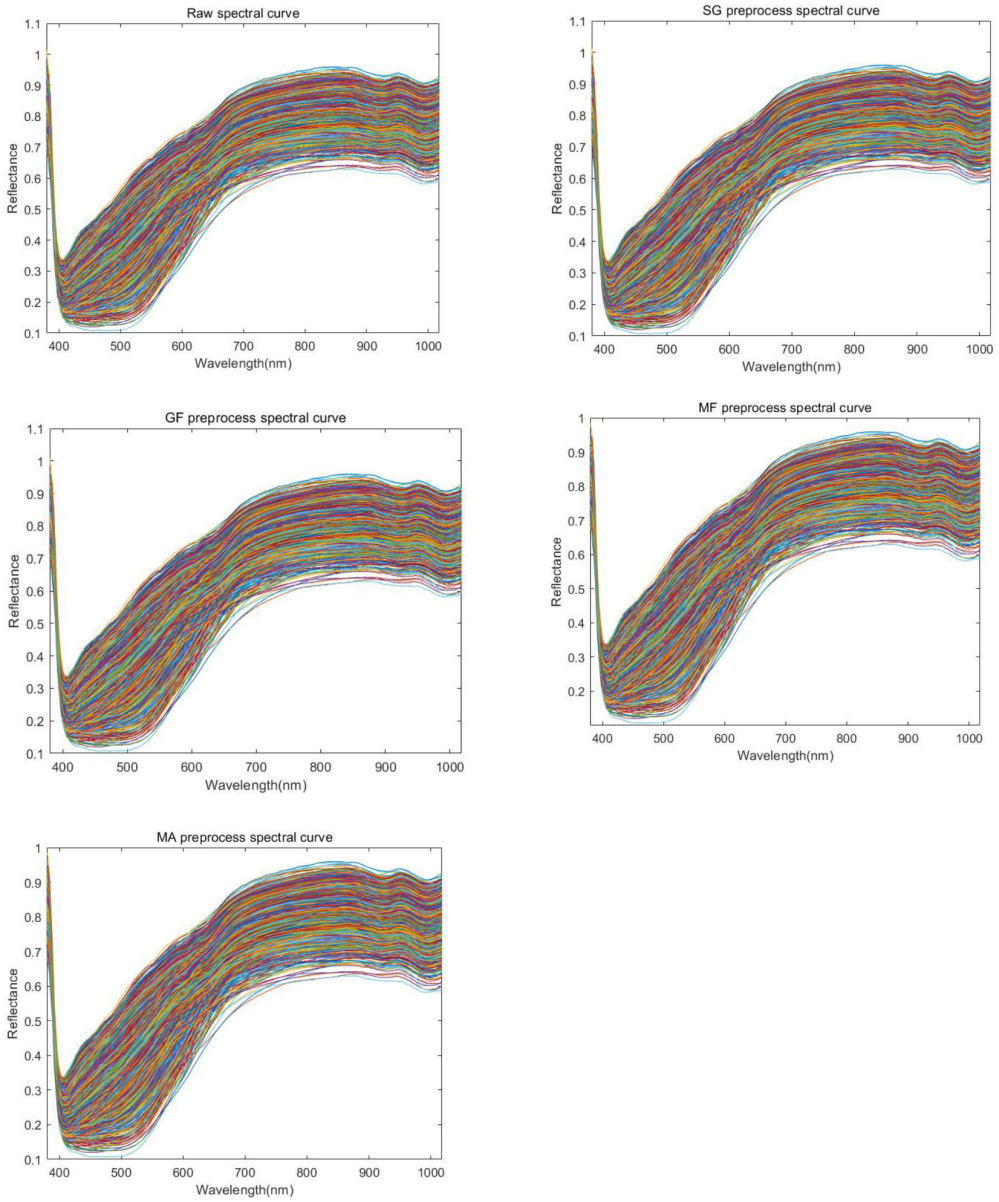
Spectral reflectance curve of maize. Representing the original image(RAW spectral curve), first-order derivative(1st Der preprocess spactral curve), second-order derivative(2nd Der preprocess spectral curve), and spectral images after preprocessing operations such as SG(SmoothingSGolay preprocess spectral curve), MF(MedianFilter preprocess spectral curve), MA(MovingAverage preprocess spectral curve), GF(GaussianFilter preprocess spectral curve), etc.

The band diagram of the original data and the four preprocessing methods is shown in **Figure 7**. Spectral curves of different colors in the figure represent different maize seeds.

### 4.2 Traditional machine learning model classification

In order to compare the effectiveness of machine learning and deep learning methods in the classification task of this study, the spectral data were classified using machine learning models and deep learning models respectively.The experimental results were obtained by averaging using the cross-ten-fold method.

In both machine learning models, after using preprocessing it was found that the preprocessing effect could not be better than the original data. Firstly, the KNN model was used for classification, and the results of k from 1 to 20 were tested under the treatments of mean processing, maximum-minimum processing and logarithmic processing, respectively, and ten experiments were conducted for each k value, and the average value was taken for the comparison of the results, and it was found that the classification effect was the best at k=17. Among the three treatments, the logarithmic treatment is the most effective and can reach 64.69% when using Mahalanobis distance as a metric, which is higher than 62.28% for the mean treatment and 58.94% for the max-min treatment. Secondly, using ELM model for classification, the number of hidden layer nodes were set to 100,150,200 and its accuracy was 80.46%, 87.06% and 83.17% respectively, so 150 was chosen as the number of hidden layer nodes for the ELM model in this study. The summary results are shown in the **Table 2**.

**Table 2:** Different machine learning results.

Model	Parameters	Accuracy rate
KNN	K value = 17 Mahalanobis distance	62.28%
ELM	Hidden layer nodes = 150	87.06%

### 4.3 Deep learning model classification

Next this study used a CNN model for classification. Firstly, the effect of different preprocessing methods on the experiment is investigated, using the original data and the data preprocessed by three different methods in one layer of convolutional CNN for comparison, respectively, it is found that the test set preprocessed by SG smoothing method has the highest accuracy, which can reach 92.03%, higher than the 90.74% of the original data, which is an enhancement of 1.29%, and all of them are far better than the classification effect of KNN, which proves that SG smoothing method for preprocessing can improve the accuracy of classification, so this study chooses SG smoothing to preprocess the original data. The preprocessing results are shown in **Table 3**.

**Table 3:** Results of different pretreatment.

Digital	Training set	Test set	Precision	Recall	F1	Time
raw data	95.33% ± 0.52%	90.74% ± 0.36%	90.57% ± 0.84%	90.25% ± 0.77%	90.41% ± 0.79%	97.32ms
SG	94.07% ± 0.34%	92.03% ± 0.25%	90.52% ± 0.21%	90.34% ± 0.22%	90.34% ± 0.22%	100.14ms
MA	94.07% ± 0.43%	91.67% ± 0.38%	90.33% ± 0.46%	90.39% ± 0.47%	90.36% ± 0.46%	104.33ms
GF	95.09% ± 0.48%	91.48% ± 0.32%	90.17% ± 0.41%	90.24% ± 0.35%	90.21% ± 0.39%	102.54ms
MF	95.14% ± 0.40%	91.30% ± 0.36%	89.87% ± 0.54%	90.12% ± 0.48%	90.01% ± 0.52%	102.37ms

Among these, metrics such as precision, recall, F1 score, and time are test set metrics. All metrics represent the average results obtained from 10-fold cross-validation. Positive and negative values indicate the difference between the average and the maximum/minimum values.

When experimenting with different feature band selection methods, it was found that the overall classification accuracy decreased with the addition of feature band selection, where the highest classification accuracy was only 85.93% using SG preprocessing and selecting feature bands using UVE, so this study used the full band for classification experiments. This operation is directly learned from the data by the machine, which reduces the influence caused by possible human operations as a way to improve the reliability of classification. Although it increases the complexity of data processing and computation, with the continuous improvement of computer computing power, the application of this method will be more widely used, and compared with the traditional method of extracting the characteristic wavelength, it reduces the manual workload, simplifies the operation steps, and only needs to be pre-processed directly to the data for classification experiments.

**Figure 8:**
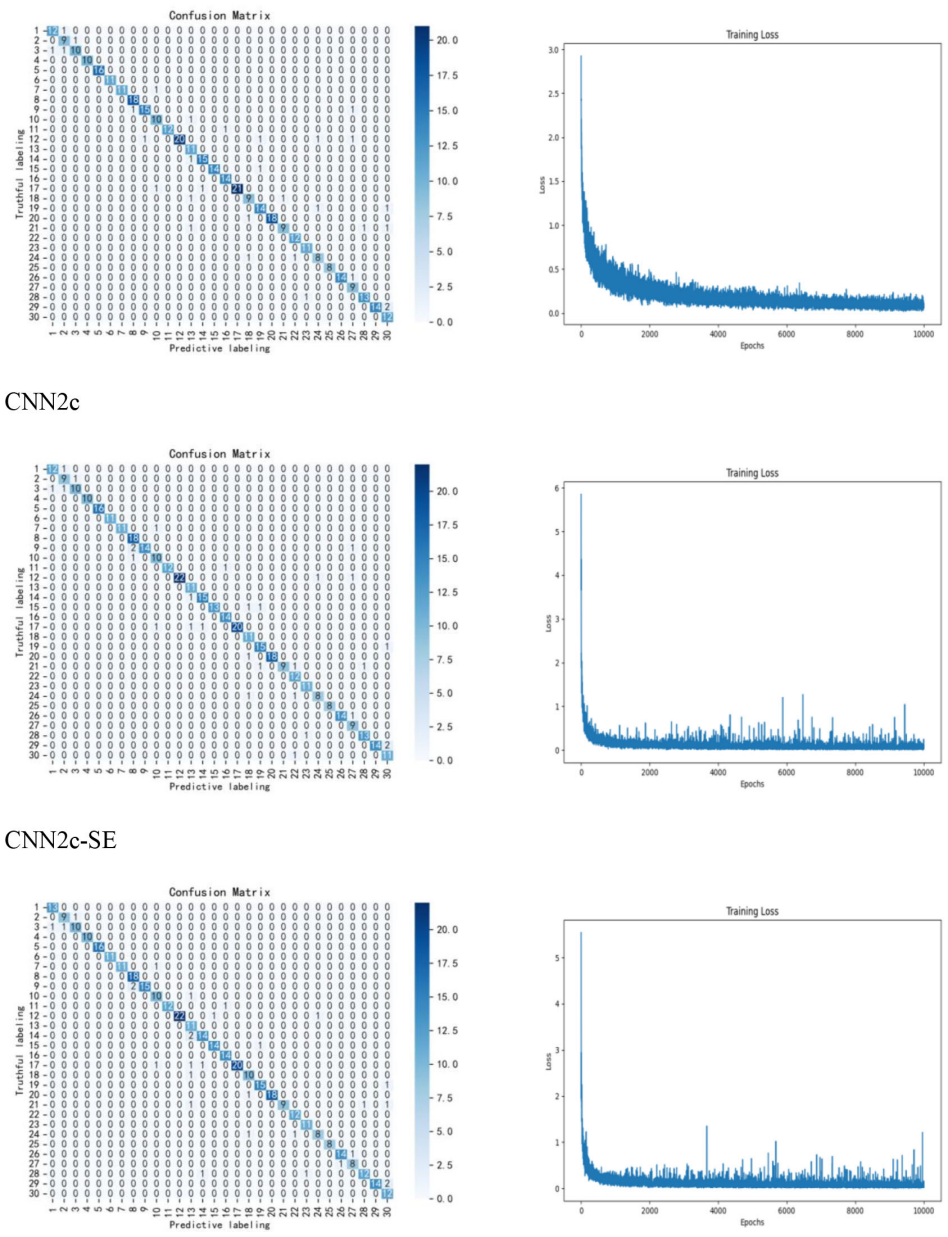
Confusion matrix and loss function.

Next, the effect of different number of convolutional layers on the experimental results was studied and compared, adding one convolutional layer and one fully connected layer to classify the preprocessed data, the accuracy of the test set increased to 93.14%, proving that there was an increase in the classification accuracy at two convolutional layers and two fully connected layers, which improved the accuracy by 1.11%. Adding another convolutional layer no longer improves the accuracy, so the CNN with two convolutional layers and two fully connected layers is finally used as the classification model. Finally, we explore the experimental effect of improving the model. In this study, the SE Block module is added to the CNN network, to systematically optimize this parameter and assess its impact, we have conducted a ablation study testing different reduction ratios (r = 4, 8, 16, 32), The results indicate that a ratio of r=16 yielded the best performance for our specific dataset and model architecture, The results are shown in **Table 4**. The accuracy of using the CNN2c-SE test set can be improved to 93.89%, which is 0.75% higher than the classification accuracy of the model before improvement, proving that the CNN2c-SE model can improve the classification accuracy of the 30 maize seeds in this study. Its confusion matrix results and loss function are shown in **Figure 8**. The ablation results are shown in **Table 5**.

**Table 4:** SE module ablation experiment results.

Ratio	Training set	Test set	Precision	Recall	F1	Time
4	95.32% ± 0.47%	92.85% ± 0.40%	91.31% ± 0.26%	90.52% ± 0.20%	90.90% ± 0.22%	100.52ms
8	95.40% ± 0.30%	93.74% ± 0.35%	94.65% ± 0.24%	93.48% ± 0.20%	94.06% ± 0.21%	102.73ms
16	95.55% ± 0.34%	93.89% ± 0.33%	94.37% ± 0.30%	93.72% ± 0.26%	94.04% ± 0.30%	105.34ms
32	95.38% ± 0.31%	92.72% ± 0.36%	92.42% ± 0.31%	93.42% ± 0.25%	92.92% ± 0.29%	110.67ms

Among these, metrics such as precision, recall, F1 score, and time are test set metrics. All metrics represent the average results obtained from 10-fold cross-validation. Positive and negative values indicate the difference between the average and the maximum/minimum values.

**Table 5:** Results of ablation experiments.

Mould	Training set	Test set	Precision	Recall	F1	Time
CNN1c	94.07% ± 0.22%	92.03% ± 0.30%	90.52% ± 0.21%	90.34% ± 0.22%	90.34% ± 0.22%	100.14ms
CNN2c	95.54% ± 0.25%	93.14% ± 0.26%	92.56% ± 0.26%	91.62% ± 0.27%	92.09% ± 0.27%	101.63ms
CNN2c-SE	95.55% ± 0.34%	93.89% ± 0.33%	94.37% ± 0.30%	93.72% ± 0.26%	94.04% ± 0.30%	105.34ms

Among these, metrics such as precision, recall, F1 score, and time are test set metrics. All metrics represent the average results obtained from 10-fold cross-validation. Positive and negative values indicate the difference between the average and the maximum/minimum values.

In this study, 30 varieties of maize seeds are taken as the research objects, and the classification effects of different models, different preprocessing methods, different feature band selection methods and the network model improvement are discussed. The results show that the classification effect when using convolutional neural network is much higher than that of KNN and ELM, and it can reach more than 90%, which proves that the classification model is feasible in this study, and the classification effect is further improved after the data are preprocessing and the network model is improved, the classification effect is further improved, while the selection of feature bands reduces the classification accuracy. It is proved that the use of full-band data and the improvement of the network in this study is feasible and can bring improvement to the research effect, therefore, it is determined that the use of full-band data and the improved CNN2c-SE model can effectively classify the 30 varieties of maize seeds in the study.

## 5 Summary

In this paper, hyperspectral imaging technology and CNN2c-SE model are used to classify 30 varieties of corn seeds. In order to explore the classification effect of the CNN model in the whole wavelength range, avoid the loss of part of the information due to the selection of special wavelengths, retain as much spectral information as possible, and simplify the manual operation to reduce the human influence, this study no longer carries out the selection of feature wavelengths, and uses all the spectral data in 320 wavelength bands to conduct the classification experiments, and the results show that the classification accuracy can reach 90.74% with the unimproved CNN model without preprocessing and without selecting the feature wavelengths. The results show that without preprocessing and without selecting feature bands, the classification accuracy of the unimproved CNN model can reach 90.74%, which is able to meet the requirements of the classification task, and is much higher than the accuracy of the machine learning model KNN (64.69%) and the accuracy of the machine learning model ELM (87.06%), and therefore, the CNN model is selected for classification. Data preprocessing by SG smoothing method and improving CNN model using CNN2c-SE model for classification can reach 93.89% accuracy, which significantly improves the accuracy of classification. Therefore, the use of CNN2c-SE can realize the rapid nondestructive detection of corn varieties under the full band.

Although our study included 30 varieties, all seeds were sourced from a single geographic region and harvest year. This could potentially limit the model’s generalizability to seeds grown under different environmental conditions which can influence spectral profiles. We addressed this in part through our cross-validation and independent batch test. Future work will explicitly incorporate multi-origin and multi-year data to build more robust models. Given the high dimensionality of full-spectrum data relative to the sample size, overfitting becomes a primary concern. We employ multiple strategies to mitigate this risk: adopting a simple convolutional neural network architecture rather than overly complex models; incorporating Dropout and L1 regularization; implementing early stopping based on a holdout validation set; and using 10-fold cross-validation to report performance metrics. These measures ensure that the reported performance is genuinely reliable and not attributable to overfitting. This study utilized the spectral range of 380–1018 nm. Future work will employ cameras with broader spectral ranges to collect data and compare the effect of different spectral ranges on maize seed variety classification accuracy, thereby enhancing experimental scalability. To minimize spectral information loss, the full spectrum was used. Subsequent studies will investigate a combined approach of full-spectrum and feature wavelength selection to balance efficiency with accuracy. And the collected data can also be used for subsequent seed quality testing to guarantee seed quality.

## Data Availability

The original contributions presented in the study are included in the article/supplementary material. Further inquiries can be directed to the corresponding author.
